# The role of school enjoyment and connectedness in the association between depressive and externalising symptoms and academic attainment: Findings from a UK prospective cohort study

**DOI:** 10.1016/j.jad.2021.08.043

**Published:** 2021-12-01

**Authors:** Tim Cadman, Amanda Hughes, Caroline Wright, José A López-López, Tim Morris, Frances Rice, George Davey Smith, Laura D Howe

**Affiliations:** a Integrative Epidemiology Unit (IEU), University of Bristol, Bristol, United Kingdom; b Population Health Science, Bristol Medical School, University of Bristol, United Kingdom; c Department of Basic Psychology & Methodology, University of Murcia, Spain; d Division of Psychological Medicine and Clinical Neurosciences, Cardiff University

**Keywords:** ALSPAC, school enjoyment, school connectedness, education, depression, externalising, ALSPAC, Avon Longitudinal Study of Parents and Children, UK, United Kingdom, IQ, Intelligence Quotient, RCT, Randomised Control Trial, GCSE, General Certificate of Secondary Education, CI, Confidence Interval, RMSEA, Root Mean Square Error of Approximation, SRMR, Standardised Root Mean Residual, CFI, Comparative Fit Index, TLI, Tucker Lewis Index

## Abstract

**Background:**

Previous research on the relationship between children's depressive and externalising symptoms, experience of school, and academic attainment is inconclusive. The aims of this study were (i) to test bidirectional associations between children's school experience and depressive and externalising symptoms at age 10-11 and 13-14, (ii) to ascertain whether school experience age 13-14 is associated with academic attainment age 16, and (iii) to test whether school experience mediates the relationship between depressive or externalising symptoms and attainment.

**Methods:**

Data was used from the Avon Longitudinal Study of Parents and Children (n=6,409). A cross-lagged model was used to investigate bidirectional associations between school experience (enjoyment and connectedness) and depression and externalising at age 10-11 and 13-14. The same framework was used to test if school experience aged 13-14 mediated associations of depressive and externalising symptoms with later attainment.

**Results:**

Depressive and externalising symptoms at 10-11 were negatively associated with school connectedness (depressive: standardised β=-0.06, CI: -0.11, 0.01; externalizing: β=-0.13, CI: -0.17, -0.08), and school enjoyment at 13-14 (depressive β=-0.04, -0.08, 0.03; externalising: β=-0.08, CI: -0.13, -0.03). School enjoyment at 13-14 was positively associated with attainment at 16 (β=0.10, CI: 0.04, 0.15), and partially mediated associations between depressive and externalising symptoms at 10-11 and attainment at 16 (depressive: proportion mediated 2.2%, CI: -1.5, 5.9; externalising: proportion mediated; 4.7%, CI: 0.7, 10.1,).

**Limitations:**

Results may be subject to residual confounding.

**Conclusions:**

School enjoyment is a potentially modifiable risk factor that may affect educational attainment of adolescents with depressive or externalising symptoms.

## Highlights

• We found that school enjoyment at age 13-14 was strongly associated with academic attainment at age 16.
• Children experiencing depressive or externalising symptoms at age 10-11 were less likely to enjoy school and feel connected to staff and teachers at age 13-14.
• We found evidence that school enjoyment partially mediated the association between earlier depressive and externalising symptoms and academic attainment. This suggests that children's experience of school could be a potentially important modifiable risk factor for low attainment in children with mental health problems.

## 1 Introduction

There is consistent evidence that children with depressive symptoms (McLeod et al., 2012; Mykletun et al., 2016; Veldman et al., 2014) and hyperactive and conduct difficulties (“Externalising” Currie and Stabile, 2006; Fletcher and Wolfe, 2008; Frazier et al., 2007; McLeod et al., 2012; Mykletun et al., 2016; Sayal et al., 2015; Veldman et al., 2014; Washbrook et al., 2013) have on average lower academic performance than unaffected peers. This is of importance because poorer educational attainment in childhood is linked with worse health and economic outcomes in adulthood (Easterbrook et al., 2016). To improve life-long outcomes for children with mental health problems it is crucial to understand the pathways between these problems and low attainment to inform potential interventions.

One potentially important mediating factor in the educational attainment of children with affective or externalising symptoms is their experience of school. Adolescent depression is associated with poorer social functioning, (Verboom et al., 2014) friendships (Goodyer et al., 1997) and academic self-concept, (Quiroga et al., 2013) which could affect how children experience school. School culture in the UK – which emphasises quiet, focused work – can also be a poor ‘fit’ for children with attentional and behavioural problems (Bailey and Thomson, 2009; Richardson et al., 2015). Children with these difficulties can elicit more negative responses from staff, have poorer relationships with peers and are more likely to be excluded from school (Henricsson and Rydell, 2004; Hoza, 2007; Parker et al., 2015).

Various aspects of the school experience have been previously defined (Libbey, 2004). “School enjoyment” describes a child's overall attitude towards school, “Connectedness” refers to their sense of belonging and feeling accepted within their school, whilst “School Climate” refers to children's experience of the relationships, norms and practices within their school (Shinde et al., 2018). Whilst these are overlapping constructs, different aspects of school experience may have different antecedents and consequences.

Studies have consistently showed that school enjoyment is prospectively associated with academic attainment (Morris, 2019; Riglin et al., 2013). For example, a recent UK study found that school enjoyment measured at age 6 was associated with academic attainment age 16 as strongly as maternal education (an indicator of socioeconomic position) (Morris, 2019). However, evidence for the relationship between school experience and depressive and externalising symptoms is less clear. A number of studies have reported positive prospective associations between different aspects of school experience and depressive and externalising symptoms (García-Moya et al., 2018; Kidger et al., 2015; Kuperminc et al., 2001; Markowitz, 2017; Resnick et al., 1997; Shochet et al., 2006; Viner et al., 2012; Wickrama and Vazsonyi, 2011). However, evidence for the converse associations between depressive/ externalising symptoms and school experience is inconsistent. Using a cross-lagged design, Lester et al. found bidirectional associations between depression and anxiety and children's feeling of connection with school, (Lester et al., 2013; n=3,459) but Shochet et al. reported the association between anxiety and depressive symptoms and school connectedness to be close to null (Shochet et al., 2006; n=2,022). A third study by Loukas et al (n=296) was too small to draw any firm conclusions (Loukas et al., 2013).

Importantly, there is emerging evidence that children's academic attainment, school experience and their socioemotional well-being can be improved by school-based interventions. For example, a recent RCT in India reported that a school-based intervention which focused on teaching problem-solving skills and engaging students and teachers in decision-making improved student's perceptions of their school and reduced rates of depressive symptoms (Shinde et al., 2018). A UK-based RCT tested an intervention focused on increasing school engagement through improving relationships between students and teachers, and actively engaging students to remodel school practices to centre on student needs. In addition to reducing bullying rates, this intervention also led to an improvement in self-reported quality of life, well-being and a reduction in psychological problems (Bonell et al., 2018).

In this current study we focused on two key aspects of school experience: school enjoyment and connectedness. We had three main aims: (i) to test the associations between depressive and externalising symptoms and school enjoyment and connectedness at two time points (ages 10-11 and ages 13-14), (ii) to explore associations between school enjoyment and connectedness at ages 13-14 and academic attainment age 16, and (iii) to test whether school enjoyment or connectedness at ages 13-14 mediates the relationship between depressive or externalising symptoms at age 10-11 and academic attainment at age 16.

## 2 Methods

### 2.1 Sample

Data was used from the Avon Longitudinal Study of Parents and Children (ALSPAC). Pregnant women resident in Avon, UK with expected dates of delivery 1st April 1991 to 31st December 1992 were invited to take part in the study. The initial number of pregnancies enrolled is 14,541 (for these at least one questionnaire has been returned or a “Children in Focus” clinic had been attended by 19/07/99). Of these initial pregnancies, there was a total of 14,676 foetuses, resulting in 14,062 live births and 13,988 children who were alive at 1 year of age. (For further details on the cohort profile, representativeness, and phases of recruitment, see Boyd et al., 2013; Fraser et al., 2013). The study website contains details of all data available through a fully searchable data dictionary and variable search tool: http://www.bristol.ac.uk/alspac/researchers/our-data/. Ethical approval for the study was obtained from the ALSPAC Ethics and Law Committee and the Local Research Ethics Committees. Informed consent for the use of data collected via questionnaires and clinics was obtained from participants following the recommendations of the ALSPAC Ethics and Law Committee at the time.

### 2.2 Measures

*Depressive symptoms.* Depressive symptoms were self-reported using the Short Mood and Feelings Questionnaire at mean ages 10.5 (T1) and 14 (T2) (Angold et al., 1995). This questionnaire contains 13 items scored 0, 1 or 2 giving a total range of 0 – 26, with higher scores indicating greater depressive symptoms.

*Externalising symptoms.* Externalising symptoms were measured via maternal report using the Conduct and Hyperactivity subscales of the Strengths and Difficulties Questionnaire at mean ages 11.5 (T1) and 13 (T2) (Goodman et al., 2000). Both subscales contain 5 items scored 0, 1 or 2 giving a total range of 0 – 10, with higher scores indicating greater difficulties. These scales were modelled as one factor in the main analysis, but additional analyses modelled the scales separately.

*School experience.* School experience was assessed via self-report at ages 11 (T1) and 14 (T2). Previous research has posited multiple overlapping constructs related to school experience, (Libbey, 2004) here we focus on two aspects: (i) enjoyment and (ii) connectedness (Kidger et al., 2015). School enjoyment items refer to the child's enjoyment of classes and school (for example “School is a place where I enjoy what I do in class”). School connectedness items ask about the child's sense of belonging at the school and how positively they believe they are viewed by others (for example “School is a place where people can depend on me”), and is closely related to concepts such as school climate (Shinde et al., 2018; Shochet et al., 2006). Higher scores indicate greater enjoyment and connection. Full details of questions are provided in Tables S2 & S4.

*Academic attainment.* Academic attainment was measured by capped General Certificate of Secondary Education (GCSE) point score at age 16 (T3), obtained via linkage to the National Pupil Database. GCSEs are qualifications taken at age 16, which represented the end of compulsory education in the UK for this cohort. Capped GCSE point scores are continuous scores representing the best 8 grades at GCSE. Each grade has a different value (for example A* which represents the highest possible grade is worth 58 points, C is worth 40 points) and these are summed to produce the total score (Education, 2015). For the majority of students the capped score will range between 0 – 464; however it is possible some may have scores up to 540 if they took more advanced qualifications than GCSE at age 16. The capped, rather than total GCSE score was chosen because it reduces the influence of scores from pupils who take more than the standard number of examinations.

*Covariates.* The following variables were identified *a priori* as confounders as they had either previously been found to or could plausibly influence depressive symptoms, externalising, school experience and attainment: child sex at birth, maternal smoking in pregnancy (yes/no), housing tenure (owner/mortgaged vs other), highest parental education (None, Vocational, O-level, A-level, Degree), self-reported material hardship (yes/no), parity, IQ and earlier school enjoyment (Huisman et al., 2010; Joinson et al., 2017). Early school enjoyment was measured by binary items at ages 5 and 6 asking the child whether they enjoy going to school. Child IQ was assessed at age 8 using a short form of the Weschler Intelligence Scale for Children - III, (Wechsler, 1992) whilst all other confounders were measured during pregnancy.

### 2.3 Statistical analysis

**Fig. 1:**
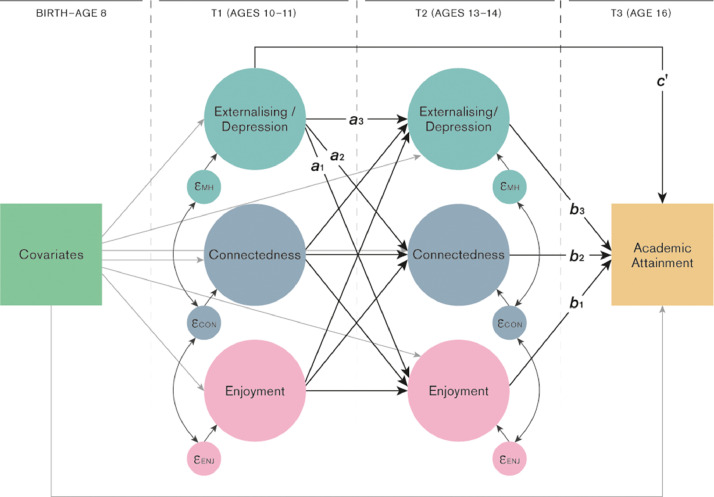
Connectedness = child's sense of connection with teachers and peers; Enjoyment = child's enjoyment of school, Academic attainment = child's GCSE point score. “Covariates” are child sex, maternal smoking in pregnancy, housing tenure, highest parental education, self-report of material hardship, parity, child IQ and school enjoyment at ages 5 and 6. Single-headed arrows represent regression pathways, double-headed arrows represent residual correlations. Paths a1-a3 and b1-b3 represent indirect effects, path c’ represents the direct effect.

The relationship between study variables was tested using structural equation modelling. Depressive symptoms, externalising, school enjoyment and school connectedness were modelled as latent variables. A cross-lagged panel design was used to test the associations between school experience and depressive/externalising symptoms at ages 10-11 and 13-14 (Fig. 1; separate models for depressive and externalising symptoms). The cross-lagged design allows the estimation of associations between multiple time points whilst controlling for within time-point correlations and the stability of constructs over time (Allen, 2017). Additional correlations were specified between the error terms of pairs of items repeated at T1 and T2. All results are presented in standardised coefficients (β) to allow comparison between estimates.

Potential mediation of the association between depressive/externalising symptoms and academic attainment via school experience was also tested within the structural equation modelling framework. Three indirect effects (Fig. 1: a_1_ * b_1_, a_2_ * b_2_ and a_3_ * b_3_) were calculated from the paths from age 10-11 depressive/externalising symptoms to age 16 attainment. The direct effect (c’) is the association between age 10-11 depressive/externalising symptoms and age 16 attainment whilst adjusting for age 13-14 depressive/externalising symptoms, enjoyment and connectedness*.*

For both depressive and externalising symptoms, an unadjusted model was tested followed by two levels of adjustment (i) a minimally adjusted model (child sex, maternal smoking in pregnancy, housing tenure, highest parental education, self-report of material hardship, parity and school enjoyment at ages 5 and 6) and (ii) a model additionally adjusted for child IQ. As previously argued, (Washbrook et al., 2013) although IQ is potentially a confounder of the relationships between depressive/externalising symptoms, school experience and attainment, it may also be on the causal pathway from earlier depressive/externalising symptoms or school experience to later attainment. If differences in IQ are (even partially) caused by earlier depressive/externalising symptoms or school experience (e.g. through lower motivation or concentration), then adjusting for IQ would bias the estimates downwards. Conversely, if IQ is purely a confounder then an IQ-adjusted model would be correct. However, given the available data it is not possible to distinguish between the two situations. The main results are presented for the minimally adjusted model, with all models reported in Supplementary Materials. Child sex was associated with most exposures and outcomes (Table S3) therefore analyses were also repeated stratified by sex. The model was estimated using Diagonal weighted least squares with robust versions of fit statistics, using the lavaan package in R version 3.53 (Rosseel, 2012).

### 2.4 Inclusion criteria, and method for addressing missing data

Missing data were imputed for all variables in the model using multiple chained equations for the sample of subjects with complete data on GCSE attainment at age 16 and at least one measure of depressive or externalising symptoms and one school experience measure at one time point. Imputation was conducted using the MICE package in R (van Buuren and Groothuis-Oudshoorn, 2011). Data were imputed separately for males and females and then combined. The imputation model contained all variables within the model and additional variables known to be predictors of missingness including earlier measurements of school enjoyment and depressive/externalising symptoms (full details of the imputation model and distribution of original and imputed data are provided in Supplementary Materials). 20 datasets were imputed and estimates combined using Rubin's rules (Rubin, 2004).

## 3 Results

### 3.1 Descriptive statistics

The mean GCSE point score was 344.67 (SD: 78.83). Mean IQ score at age 8 was 103.88 (SD: 16.42), indicating scores slightly above the standardised average of 100. Distributions of school experience variables were skewed towards a positive school experience, and distributions of depressive symptoms and externalising towards lesser symptoms (Table S2).

Correlations between school enjoyment and connection were 0.73 (95% confidence interval (CI): 0.72, 0.74) at ages 10-11 and 0.58 (CI: 0.56, 0.60) at ages 13-14. Correlations between depressive and externalising symptoms were 0.24 (CI 0.20, 0.27) at ages 10-11 and 0.21 (CI: 0.17, 0.25) at ages 13-14.

Descriptive statistics suggest that the analysis sample was of higher average socioeconomic position compared to the full ALSPAC sample (Table S2). For example, mean GCSE point score in the full ALSPAC sample was 318.94 (SD: 93.95) compared to 344.67 (SD: 78.33) in the analysis sample; similarly average maternal age at birth in full ALSPAC sample was 28.64 (SD: 4.86) vs 29.31 (SD: 4.55) in the analysis sample.

### 3.2 Model fit

Fit for the latent models of school experience, depressive and externalising symptoms was good (average fit statistics across imputations, depressive symptoms model RMSEA = 0.04, SRMR = 0.04, CFI = 0.99, TLI = 0.99; externalising model RMSEA = 0.06, SRMR = 0.06, CFI = 0.97, TLI = 0.97). Loadings were moderate to high on all items (0.43 – 0.95), apart from one item (“School is a place where I get excited about the work I do”) with a very low loading of 0.01 (Tables S2 - S4). This item was retained in the model to allow comparison with previous findings from the ALSPAC cohort.

### 3.3 Main findings

**Fig. 2:**
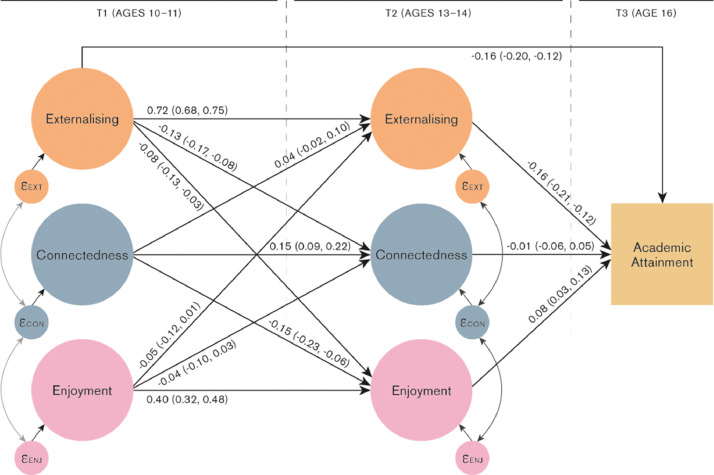
Connectedness = child's sense of connection with teachers and peers; Enjoyment = child's enjoyment of school, Academic attainment = child's GCSE point score. “Covariates” are child sex, maternal smoking in pregnancy, housing tenure, highest parental education, self-report of material hardship, parity, child IQ and school enjoyment at ages 5 and 6. Residual covariances and covariates included in model but omitted from figure for clarity.

**Fig. 3:**
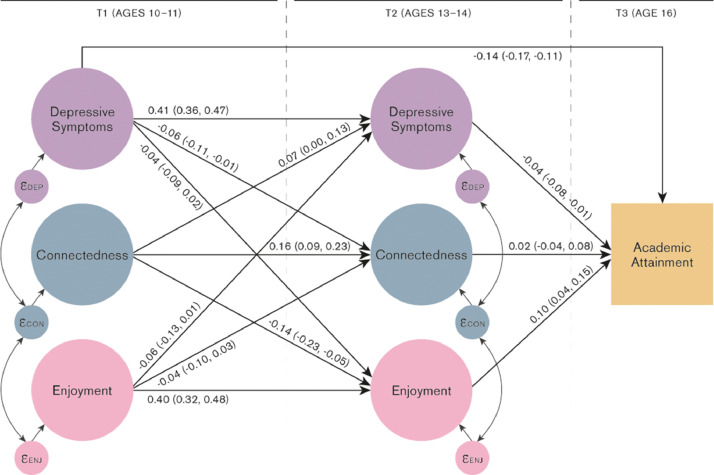
Connectedness = child's sense of connection with teachers and peers; Enjoyment = child's enjoyment of school, Academic attainment = child's GCSE point score. “Covariates” are child sex, maternal smoking in pregnancy, housing tenure, highest parental education, self-report of material hardship, parity, child IQ and school enjoyment at ages 5 and 6. Residual covariances and covariates included in model but omitted from figure for clarity.

The key results are shown in Figs. 2 and 3 and Tables S9 and S10.

*Stability of school enjoyment, depressive and externalising symptoms over time.* School enjoyment showed moderate stability between ages 10-11 and 13-14 (β = 0.40, CI: 0.32, 0.48) whilst connectedness showed lower stability (β = 0.15, CI: 0.09, 0.22). Externalising showed the highest stability between time points (β = 0.72, CI: 0.68, 0.75) whilst the stability of depressive symptoms was lower (β = 0.41, CI: 0.36, 0.47).

*Associations between school experience and externalising symptoms at ages 10-11 and 13-14 (*Fig. 2*, Table S9).* There was weak evidence of a negative association between school enjoyment aged 10-11 and externalising at age 13-14 (β = -0.05, CI: -0.12, 0.01). We also found weak evidence for a positive association between school connectedness age 10-11 and externalising age 13-14, though confidence intervals crossed the null (β = 0.04, CI: -0.02, 0.10).

There was clearer evidence of negative associations between externalising age 10-11 and school experience at 13-14 (externalising to enjoyment: β = -0.08, CI: -0.13, -0.03; externalising to connectedness: β = -0.13, CI: -0.17, -0.08). Modelling hyperactivity and conduct problems separately indicated stronger associations with school experience age 13-14 for conduct problems than for hyperactivity/impulsivity at 10-11 (Tables S17 & S18). Associations were similar for males and females, except for that of age 10-11 externalising symptoms with age 13-14 enjoyment which was larger for boys than girls (Tables S12 & S13). Associations did not differ notably when additionally adjusted for IQ.

*Associations between school experience and depressive symptoms at ages 10-11 and 13-14 (*Fig. 3*, Table S10).* A similar pattern of results was found for depressive symptoms. There was weak evidence of a negative association between school enjoyment age 10-11 and depressive symptoms age 13-14 (β = -0.06, CI: -0.13, 0.01). We also found evidence for a positive association between connectedness age 10-11 and depressive symptoms age 13-14 (β = 0.07, CI: 0.00, 0.13).

Negative associations were observed between depressive symptoms age 10-11 and school experience age 13-14 (depressive symptoms to enjoyment: β = -0.04, CI: -0.09, 0.02; depressive symptoms to connectedness: β = -0.06, CI: -0.11, -0.01). Analyses stratified by sex indicated that associations were larger for girls than boys (Tables S13 & S14). There was only minimal attenuation of associations after additionally adjusting for IQ.

*Associations between depressive/externalising symptoms age 13-14 and academic attainment age 16 (*Figs. 2*& 3, Tables S9 & S10).* Externalising symptoms at both ages 10-11 and 13-14 were negatively associated with attainment at age 16 (age 10-11: β = -0.16, CI: -0.21, -0.10; age 13-14: β = -0.16, CI: -0.20, -0.12), as were depressive symptoms (age 10-11: β = -0.14, CI: -0.17, -0.11; age 13-14: β = -0.04, CI: -0.08, -0.01). These associations were attenuated by approximately 25-50% once adjusting for IQ.

*Associations between school experience age 13-14 and academic attainment age 16 (*Figs. 2*& 3, Tables S9 & S10).* After adjusting for potential confounders, school enjoyment at age 13-14 was positively associated with attainment at age 16 (β = 0.08, CI: 0.03, 0.13); but connectedness was not (β = -0.01, CI: -0.06, 0.05). Adjusting for child IQ did not substantially change these estimates. The association between school enjoyment and attainment was notably larger for girls than boys (girls β = 0.15, CI: 0.09, 0.21; boys β = 0.03, 95% CI: -0.14, 0.20)

*Does school experience mediate the association between depressive/externalising symptoms and attainment? (*Figs. 2*& 3, Tables S9 & S10)* There was evidence that school enjoyment partially mediated the association between externalising and depressive symptoms ages 10-11 and attainment at age 16 (Fig. 1, path a_1_ * b_1_). The proportion of the association between age 10-11 externalising and age 16 attainment mediated via age 13-14 enjoyment was 4.7% (CI: 0.7, 10.1); for depressive symptoms to attainment the proportion mediated via enjoyment was 2.2%, (CI: -1.5, 5.9). However, there was minimal evidence of mediation via school connectedness (Fig. 1, path a_2_ * b_2_, proportion mediated externalising to attainment via connectedness -0.5%, CI: -5.2, 4.3; proportion mediated depressive symptoms to attainment via connectedness 0.6%, CI: -1.6, 2.8).

## 4 Discussion

The aims of this study were: (i) to test associations between depressive/externalising symptoms and school experience at two time points in secondary school (ages 10-11 and ages 13-14), (ii) to explore whether school experience at ages 13-14 was associated with academic attainment age 16, and (iii) to test whether school experience at ages 13-14 mediated the relationship between depressive/externalising symptoms at age 10-11 and academic attainment at age 16.

Results support negative associations between depressive and externalising symptoms at age 10-11 and school connectedness at age 13-14. There was weaker evidence for associations between depressive and externalising symptoms age 10-11 and school enjoyment age 13-14. We found some evidence for associations between school experience age 10-11 and depressive and externalising symptoms age 13-14, though confidence intervals crossed the null. School enjoyment at age 13-14 was positively associated with attainment at age 16, and partially mediated the relationship between earlier depressive and externalising symptoms and attainment. Although the magnitude of associations was modest, our findings suggest that children with symptoms of depression, conduct problems and inattention/hyperactivity may find school less enjoyable after the transition to secondary school, which in turn affect their later academic attainment.

### 4.1 Externalising and school experience

The negative associations between externalising symptoms at age 10-11 and connectedness aged 13-14 indicates that an aspect of school experience most affected for children with externalising difficulties is their relationships with teachers and pupils. This complements existing findings that externalising negatively impacts on peer relations, (Henricsson and Rydell, 2004; Hoza, 2007) which may in turn may be one pathway to elevated levels of depression (Patterson and Stoolmiller, 1991; Powell et al., 2020). We found some evidence that conduct problems rather than hyperactivity were more strongly associated with subsequent school enjoyment; however further studies are needed to replicate this finding.

Reduced school enjoyment and connectedness for children with attentional and behavioural problems likely results from a complex interplay of individual and societal factors. School culture in the UK rewards quiet, focused and obedient behaviour, (Bailey and Thomson, 2009) and can be a particularly poor fit for children with attentional and behavioural difficulties (Richardson et al., 2015). Children entering secondary school with these difficulties are likely to struggle to meet many of the demands of school which in turn could create feedback loops (Keshavarz et al., 2010). Their behaviours may elicit a negative reaction from peers and teachers (Henricsson and Rydell, 2004; Hoza, 2007) which could result in an escalation of behaviours, leading to an increased risk of exclusion and absence, (Hughes et al., 2019; Parker et al., 2015) low mood and poor academic outcomes (Lüftenegger et al., 2016; Riglin et al., 2013; Tze et al., 2016).

### 4.2 Depressive symptoms and school experience

Similarly to externalising, children suffering depressive symptoms at the transition to secondary school were less likely to feel connected to teachers and peers at age 13-14. This fits with previous findings that depression is associated with impaired relationships (Quiroga et al., 2013; Verboom et al., 2014). The association between depressive symptoms and school enjoyment may also reflect a cognitive bias towards negative evaluation amongst children with high levels of depressive symptoms (Cole et al., 1999). We also found a small positive association between school connectedness age 10-11 and depression age 13-14. This finding was contrary to expectations and we were unable to adequately explain it. It is possible that was a chance finding that would not be replicated by further studies using different samples.

### 4.3 School experience, externalising/depressive symptoms and attainment

Consistent with previous research, (Lüftenegger et al., 2016; Morris, 2019; Riglin et al., 2013; Tze et al., 2016) we found a positive association between school enjoyment at age 13-14 and attainment at age 16, after controlling for depressive/externalising symptoms and potential confounders. However we found close to no association between connectedness age 13-14 and attainment age 16. One explanation of this difference is that two of the three items relating to enjoyment specifically ask about enjoying work, which may be more relevant to future attainment than relationships with teachers and peers. We also found notable sex differences, with a stronger association between school enjoyment and academic outcomes for boys than girls. Our finding that school enjoyment partially mediates the relationship between depressive/externalising symptoms and academic attainment suggests children's experience of school may be a potentially risk factor, although the proportion mediated was small.

Importantly, there is evidence that school enjoyment and connectedness is modifiable. Whilst there is limited evidence for the effectiveness of individual interventions to improve children's emotional well-being, (Caldwell et al., 2019; Richardson et al., 2015) trials using whole-school approaches in the US, UK and India have all shown promising results (Battistich et al., 1997; Bonell et al., 2018; Shinde et al., 2018). However, further high-quality trials are needed.

## 5 Limitations

This was a large prospective cohort study with repeated data on depressive and externalising symptoms and school experience along with linked educational data. The study design attempted to ascertain the direction of relationships and we were able control for some known confounders including child IQ. Nevertheless, as an observational study, results will be subject to residual confounding, selection bias and may suffer from reverse causation. Attrition in ALSPAC is high and associated with sex and socioeconomic factors, (Howe et al., 2013) and whilst multiple imputation was used to mitigate resultant bias, our imputed sample remained of higher socioeconomic position than the full ALSPAC sample. Though externalising and depressive symptoms often co-occur they were modelled separately due to the limited sample size and to minimise model complexity. Future research based on larger samples may be able to model these relationships together, and test for hypothesised “cascade effects”, e.g. where early behavioural problems lead to poor attainment and subsequent depression (Blain-Arcaro and Vaillancourt, 2017; Burt and Roisman, 2010).

## 6 Summary

In summary, we found evidence that depressive and externalising symptoms were prospectively associated with school connectedness, with weaker evidence for other pathways. School enjoyment was associated with academic attainment, and may partially mediate associations between depressive and externalising symptoms and later academic attainment, indicating a possible avenue for intervention.

## Funding

The UK Medical Research Council and Wellcome (Grant ref: 217065/Z/19/Z) and the University of Bristol provide core support for ALSPAC. This publication is the work of the authors and will serve as guarantors for the contents of this paper. A comprehensive list of grants funding is available on the ALSPAC website (http://www.bristol.ac.uk/alspac/external/documents/grant-acknowledgements.pdf); This research was specifically funded by the UK Medical Research Council (MR/M020894/1) and the Health Foundation's Social and Economic Value of Health Programme (Grant ID: 807293). The Medical Research Council (MRC) and the University of Bristol support the MRC Integrative Epidemiology Unit (MC_UU_00011/1). TC received funding from the European Union's Horizon 2020 research and innovation programme under grant agreement No 733206, LIFE-CYCLE project. AH received funding from the Health Foundation's Social and Economic Value of Health programme, grant number: 807293. LDH is supported by a Career Development Award from the UK Medical Research Council (MR/M020894/1). This work is part of a project entitled ‘social and economic consequences of health: causal inference methods and longitudinal, intergenerational data’, which is part of the Health Foundation's Social and Economic Value of Health Programme (Grant ID: 807293). The Health Foundation is an independent charity committed to bringing about better health and health care for people in the UK. JALL was supported by the UK Economic and Social Research Council [grant number ES/P00881X/1]. Dr Wright is funded by a Cancer Research UK Population Research Postdoctoral Fellowship (C60153/A23895). GDS works in the Medical Research Council Integrative Epidemiology Unit at the University of Bristol [MC_UU_00011/1].

## Declaration of Competing Interest

The authors declare that they have no known competing financial interests or personal relationships that could have appeared to influence the work reported in this paper.

## Appendix Supplementary materials

Image, application 1
