# Supplementary material for: The role of school enjoyment and connectedness in the association between depressive and externalising symptoms and academic attainment: Findings from a UK prospective cohort study
Source: J Affect Disord. 2021 Dec 1;295:974–80. doi: 10.1016/j.jad.2021.08.043 (PMC8572763; doi:10.1016/j.jad.2021.08.043)
Supplement: Supplementary file 1 [file mmc1.docx]

**Supporting information**

**Missing data and imputation**

Table S1 shows the available data for all covariates, mental health, school experience and GCSE data. Complete data on externalising, school experience, attainment and covariates were available for n = 944 (44% males; 56% females); for depressive symptoms plus all other variables complete data was available for n = 965 (43% males, 57% females).

Table S1. Available data

|  | **Time point** | | | | | | |
| --- | --- | --- | --- | --- | --- | --- | --- |
|  | **Pregnancy** | **Age 5** | **Age 6** | **Age 8** | **Age 10 -11** | **Age 13 - 14** | **Age 16** |
| Externalising |  |  |  |  | 6,700 | 6,587 |  |
| Depression |  |  |  |  | 7,358 | 6,015 |  |
| School enjoyment |  | 7,138 | 6,266 |  | 7,615 | 4,652 |  |
| School connectedness |  |  |  |  | 7,366 | 3,886 |  |
| Academic attainment |  |  |  |  |  |  | 10,874 |
| Child IQ |  |  |  | 7,342 |  |  |  |
| Child sex | 15,032 |  |  |  |  |  |  |
| Maternal smoking in pregnancy | 13,340 |  |  |  |  |  |  |
| Housing tenure | 13,482 |  |  |  |  |  |  |
| Parental education | 10,650 |  |  |  |  |  |  |
| Material hardship | 12,146 |  |  |  |  |  |  |
| Parity | 13,106 |  |  |  |  |  |  |
| Maternal age at birth | 12,058 |  |  |  |  |  |  |

IQ = Intelligence Quotient

The predictive model contained all exposures, outcomes and covariates described in the methods and additional variables which are known predictors of missingness (maternal smoking in pregnancy, maternal post-natal depression) as well as measures of SDQ internalising and externalising at ages 6 and 8, and six measurements of depressive symptoms (MFQ) between the ages of 16 to 24. Imputation was carried out separately for males and females and combined. Table S1 lists the percentage imputed for each variable and descriptive statistics for the original and imputed data. Results from the structural equation model based on unimputed data are at the end of Supplementary Materials (Tables S13 & S14). Results based on imputed data from this model were compared to unimputed data and pattern of results was similar.

Table S2. Comparison of distributions for imputed data, unimputed study sample and full ALSPAC sample

|  |  | Mean (SD) for continuous variables / % for categorical variables | | |
| --- | --- | --- | --- | --- |
|  | Percent imputed | Imputed data | Observed data (study sample) | Observed data (full ALSPAC sample) |
| n |  | 6,409 |  |  |
| G5: Child has often had temper tantrums or hot tempers in the last six months (%) | 16 |  |  |  |
| Not true |  | 57 | 60.7 | 60.2 |
| Somewhat true |  | 31.7 | 30.8 | 31 |
| Certainly true |  | 11.3 | 8.5 | 8.8 |
| G7: Child has been generally obedient, usually does what adults request (%) | 16 |  |  |  |
| Certainly true |  | 57 | 60.2 | 59.8 |
| Somewhat true |  | 37.9 | 36.6 | 36.7 |
| Not true |  | 5 | 3.1 | 3.6 |
| G12: Child has often fought with other children or bullied them in the last six months = Certainly / Somewhat true (%) | 17 | 8.6 | 5.2 | 5.8 |
| G18: Child has often lied or cheated in the last six months = Certainly / Somewhat true (%) | 17 | 20.1 | 15.6 | 16.6 |
| G22: Child has stolen from home/school/elsewhere in the last six months = Certainly / Somewhat true (%) | 17 | 5.1 | 1.9 | 2.1 |
| G2: Child has been restless/overactive/cannot sit still for long in the last six months (%) | 17 |  |  |  |
| Not true |  | 69.5 | 73.9 | 73.2 |
| Somewhat true |  | 22.4 | 21 | 20.9 |
| Certainly true |  | 8.2 | 5.1 | 5.9 |
| G10: Child has been constantly fidgeting or squirming in the last six months (%) | 16 |  |  |  |
| Not true |  | 74.2 | 78.7 | 77.7 |
| Somewhat true |  | 18.4 | 16.7 | 17.2 |
| Certainly true |  | 7.3 | 4.6 | 5 |
| G15: Child has been easily distracted, concentration wandered in the last six months (%) | 16 |  |  |  |
| Not true |  | 52.2 | 55.5 | 53.2 |
| Somewhat true |  | 34.6 | 34.5 | 35.2 |
| Certainly true |  | 13.1 | 10 | 11.6 |
| G21: Child has thought things out before acting in the last six months (%) | 17 |  |  |  |
| Certainly true |  | 24.3 | 25.2 | 24.8 |
| Somewhat true |  | 61.9 | 64.6 | 63.6 |
| Not true |  | 13.8 | 10.3 | 11.6 |
| G25: Child has seen tasks through to the end, had good attention span in the last six months (%) | 16 |  |  |  |
| Certainly true |  | 39.7 | 41.8 | 40.4 |
| Somewhat true |  | 45.6 | 46.9 | 47 |
| Not true |  | 14.7 | 11.3 | 12.6 |
| I7: Teenager is generally obedient, usually does what adults request (%) | 18 |  |  |  |
| Certainly true |  | 52.8 | 57.4 | 56.5 |
| Somewhat true |  | 39.9 | 39 | 39.4 |
| Not true |  | 7.2 | 3.7 | 4.1 |
| I5: Teenager has often had temper tantrums or hot tempers (%) | 18 |  |  |  |
| Not true |  | 54.2 | 58.9 | 58.2 |
| Somewhat true |  | 32.3 | 32.4 | 32.6 |
| Certainly true |  | 13.5 | 8.7 | 9.3 |
| I12: Teenager often fights or bullies other children/teenagers = Certainly / Somewhat true (%) | 19 | 9.1 | 3.8 | 4.4 |
| I18: Teenager often lies or cheats = Certainly / Somewhat true (%) | 19 | 21.2 | 14.8 | 15.7 |
| I22: Teenager steels from home, school, elsewhere = Certainly / Somewhat true (%) | 19 | 7.6 | 2.5 | 2.7 |
| I21: Teenager thinks things out before acting (%) | 19 |  |  |  |
| Certainly true |  | 23.1 | 24.7 | 24.2 |
| Somewhat true |  | 60.7 | 64.4 | 64 |
| Not true |  | 16.2 | 10.9 | 11.8 |
| I25: Teenager sees tasks through to end, has good attention span (%) | 18 |  |  |  |
| Certainly true |  | 38 | 41 | 39.9 |
| Somewhat true |  | 46.1 | 47.4 | 47.5 |
| Not true |  | 15.9 | 11.6 | 12.6 |
| I2: Teenager has been restless, overactive and can't stay still for long (%) | 18 |  |  |  |
| Not true |  | 62.5 | 68 | 67.5 |
| Somewhat true |  | 27.5 | 26.3 | 26.4 |
| Certainly true |  | 10 | 5.6 | 6.2 |
| I10: Teenager is constantly fidgeting or squirming (%) | 19 |  |  |  |
| Not true |  | 71.8 | 77.8 | 76.8 |
| Somewhat true |  | 19.2 | 17.9 | 18.3 |
| Certainly true |  | 9 | 4.3 | 4.9 |
| I15: Teenager is easily distracted, concentration wanders (%) | 19 |  |  |  |
| Not true |  | 45.9 | 50 | 48.6 |
| Somewhat true |  | 37.4 | 38.2 | 38.1 |
| Certainly true |  | 16.7 | 11.8 | 13.3 |
| Felt miserable (%) | 16 |  |  |  |
| Not at all |  | 41.6 | 45.8 | 46.1 |
| Sometimes |  | 47.6 | 48.9 | 48.3 |
| True |  | 10.8 | 5.3 | 5.6 |
| Not enjoyed anything = (Sometimes) True (%) | 16 | 18.7 | 13.2 | 13.8 |
| Felt tired, sat around (%) | 16 |  |  |  |
| Not at all |  | 53.9 | 58.5 | 58 |
| Sometimes |  | 36.6 | 36.8 | 37.1 |
| True |  | 9.5 | 4.7 | 4.9 |
| Felt very restless (%) | 16 |  |  |  |
| Not at all |  | 47.4 | 50.7 | 50.3 |
| Sometimes |  | 41.2 | 41 | 40.6 |
| True |  | 11.4 | 8.3 | 9.1 |
| Felt I was no good (%) | 16 |  |  |  |
| Not at all |  | 76.6 | 84.5 | 83.9 |
| Sometimes |  | 14.2 | 12.6 | 13.1 |
| True |  | 9.2 | 2.9 | 3 |
| Cried a lot (%) | 16 |  |  |  |
| Not at all |  | 75.1 | 81.9 | 82.2 |
| Sometimes |  | 17.4 | 15.7 | 15.5 |
| True |  | 7.5 | 2.4 | 2.3 |
| Found it hard to think (%) | 16 |  |  |  |
| Not at all |  | 42.3 | 46.2 | 44.7 |
| Sometimes |  | 46.1 | 47.9 | 48.7 |
| True |  | 11.7 | 6 | 6.6 |
| Hated myself (%) | 16 |  |  |  |
| Not at all |  | 78.7 | 86.8 | 86.7 |
| Sometimes |  | 13.5 | 11 | 10.8 |
| True |  | 7.8 | 2.2 | 2.5 |
| Bad person = (Sometimes) True (%) | 16 | 15.8 | 10 | 10.8 |
| Felt lonely (%) | 16 |  |  |  |
| Not at all |  | 61.2 | 67.9 | 67.4 |
| Sometimes |  | 28.9 | 28.8 | 28.9 |
| True |  | 9.9 | 3.3 | 3.7 |
| Nobody loved me (%) | 16 |  |  |  |
| Not at all |  | 79.9 | 88.1 | 87.8 |
| Sometimes |  | 12.2 | 10 | 10.1 |
| True |  | 7.9 | 1.9 | 2.1 |
| Not as good as others (%) | 16 |  |  |  |
| Not at all |  | 66.9 | 74.2 | 73.6 |
| Sometimes |  | 23.1 | 22.5 | 22.8 |
| True |  | 9.9 | 3.3 | 3.5 |
| Did everything wrong = (Sometimes) True (%) | 16 | 22 | 14.7 | 15.4 |
| Teenager felt miserable or unhappy in the last two weeks (%) | 27 |  |  |  |
| Not at all |  | 30.9 | 37.5 | 37.4 |
| Sometimes |  | 46.3 | 53.6 | 53.7 |
| True |  | 22.9 | 8.8 | 8.8 |
| Teenager didn't enjoy anything at all in the last two weeks = (Sometimes) True (%) | 27 | 31.6 | 19.2 | 20 |
| Teenager felt so tired he/she just sat around and did nothing in the last two weeks (%) | 27 |  |  |  |
| Not at all |  | 40.7 | 48.3 | 47.8 |
| Sometimes |  | 39.9 | 44.4 | 44.9 |
| True |  | 19.4 | 7.2 | 7.3 |
| Teenager was very restless in the last two weeks (%) | 27 |  |  |  |
| Not at all |  | 40.6 | 47.5 | 46.4 |
| Sometimes |  | 39.1 | 43.3 | 44.1 |
| True |  | 20.4 | 9.2 | 9.5 |
| Teenager felt he/she was no good any more in the last two weeks (%) | 27 |  |  |  |
| Not at all |  | 64.3 | 79.3 | 78.9 |
| Sometimes |  | 15.8 | 16.1 | 16.4 |
| True |  | 19.9 | 4.5 | 4.7 |
| Teenager cried a lot in the last two weeks = (Sometimes) True (%) | 27 | 33.9 | 19.6 | 19.6 |
| Teenager found it hard to think properly or concentrate in the last two weeks (%) | 27 |  |  |  |
| Not at all |  | 35.9 | 43.4 | 42.8 |
| Sometimes |  | 40.3 | 46.4 | 46.8 |
| True |  | 23.8 | 10.2 | 10.4 |
| Teenager hated him/herself in the last two weeks (%) | 27 |  |  |  |
| Not at all |  | 66.7 | 82.4 | 82.1 |
| Sometimes |  | 13.9 | 13.2 | 13.3 |
| True |  | 19.3 | 4.4 | 4.7 |
| Teenager was a bad person in the last two weeks (%) | 27 |  |  |  |
| Not at all |  | 66.3 | 81 | 80.4 |
| Sometimes |  | 16.5 | 16.2 | 16.7 |
| True |  | 17.3 | 2.9 | 2.9 |
| Teenager felt lonely in the last two weeks (%) | 27 |  |  |  |
| Not at all |  | 55.9 | 69.1 | 68.6 |
| Sometimes |  | 22.2 | 24.8 | 25 |
| True |  | 21.9 | 6.2 | 6.5 |
| Teenager thought nobody really loved him/her in the last two weeks (%) | 27 |  |  |  |
| Not at all |  | 67.5 | 83.4 | 83 |
| Sometimes |  | 13 | 12.7 | 12.9 |
| True |  | 19.5 | 4 | 4.1 |
| Teenager thought he/she could never be as good as other kids in the last two weeks (%) | 27 |  |  |  |
| Not at all |  | 59.4 | 73.4 | 73.1 |
| Sometimes |  | 19.1 | 20.6 | 20.8 |
| True |  | 21.5 | 6 | 6.2 |
| Teenager did everything wrong in the last two weeks (%) | 27 |  |  |  |
| Not at all |  | 65.6 | 81.3 | 80.8 |
| Sometimes |  | 16.5 | 15.9 | 16 |
| True |  | 17.9 | 2.9 | 3.2 |
| Child's school is a place where they get on well with the other pupils (%) | 8 |  |  |  |
| (Strongly) Disagree |  | 5.5 | 3.9 | 4.2 |
| Agree |  | 29.6 | 29.1 | 28.5 |
| Strongly agree |  | 64.8 | 67 | 67.3 |
| Child's school is a place where other pupils accept them for who they are (%) | 9 |  |  |  |
| Strongly disagree |  | 2.8 | 1.9 | 2.2 |
| Disagree |  | 5 | 4 | 4.1 |
| Agree |  | 30.8 | 30.8 | 30.9 |
| Strongly agree |  | 61.4 | 63.3 | 62.9 |
| Child's school is a place where other pupils are very friendly (%) | 8 |  |  |  |
| (Strongly) Disagree |  | 7.2 | 5.2 | 5.7 |
| Agree |  | 37.4 | 37.4 | 37 |
| Strongly agree |  | 55.4 | 57.4 | 57.3 |
| Child's school is a place where people can depend upon them (%) | 9 |  |  |  |
| (Strongly) Disagree |  | 6.8 | 5.1 | 5.3 |
| Agree |  | 37.3 | 37.1 | 37.1 |
| Strongly agree |  | 56 | 57.8 | 57.6 |
| Child's school is a place where people trust them (%) | 9 |  |  |  |
| (Strongly) Disagree |  | 5.8 | 4 | 4.4 |
| Agree |  | 34.5 | 34.2 | 34 |
| Strongly agree |  | 59.8 | 61.8 | 61.7 |
| Child's school is a place where they know people think a lot of them (%) | 10 |  |  |  |
| Strongly disagree |  | 5.5 | 3.9 | 4.1 |
| Disagree |  | 13.3 | 12.3 | 12.5 |
| Agree |  | 50 | 51.5 | 51.2 |
| Strongly agree |  | 31.2 | 32.2 | 32.2 |
| Child's school is a place where they have a lot of fun (%) | 8 |  |  |  |
| Strongly disagree |  | 3.7 | 2.5 | 2.9 |
| Disagree |  | 8.4 | 7.4 | 7.2 |
| Agree |  | 42.9 | 43.5 | 43.1 |
| Strongly agree |  | 45 | 46.6 | 46.8 |
| Child's school is a place where they really like to go each day (%) | 8 |  |  |  |
| Strongly disagree |  | 5.6 | 4.5 | 4.8 |
| Disagree |  | 13.3 | 12.5 | 12.2 |
| Agree |  | 59 | 60.4 | 59.6 |
| Strongly agree |  | 22.1 | 22.6 | 23.3 |
| Child's school is a place where they get excited about the work they do (%) | 9 |  |  |  |
| Strongly disagree |  | 12.4 | 11 | 11.4 |
| Disagree |  | 32.4 | 32.3 | 31.6 |
| Agree |  | 43.8 | 45.3 | 45.2 |
| Strongly agree |  | 11.4 | 11.5 | 11.8 |
| Child's school is a place where they enjoy what they do in class (%) | 08 |  |  |  |
| Strongly disagree |  | 3.4 | 2.4 | 2.4 |
| Disagree |  | 10 | 9 | 9.2 |
| Agree |  | 49.2 | 50 | 50.1 |
| Strongly agree |  | 37.4 | 38.6 | 38.3 |
| Respondent's school is a place where they get on well with other pupils in (%) | 29 |  |  |  |
| (Strongly) Disagree |  | 19.8 | 5 | 5.2 |
| Agree |  | 51.6 | 62.6 | 61.9 |
| Strongly agree |  | 28.6 | 32.5 | 32.9 |
| Respondent's school is a place where other pupils accept them (%) | 31 |  |  |  |
| Strongly disagree |  | 17.2 | 2.6 | 2.7 |
| Disagree |  | 10.2 | 10.1 | 10.3 |
| Agree |  | 52.6 | 64.4 | 63.7 |
| Strongly agree |  | 20 | 22.8 | 23.4 |
| Respondent's school is a place where other pupils are very friendly (%) | 30 |  |  |  |
| Strongly disagree |  | 17.4 | 2.5 | 2.5 |
| Disagree |  | 11.6 | 11.9 | 11.9 |
| Agree |  | 52.7 | 64.7 | 64.3 |
| Strongly agree |  | 18.3 | 20.8 | 21.3 |
| Respondent's school is a place where people can depend on them (%) | 34 |  |  |  |
| (Strongly) Disagree |  | 19 | 5.9 | 5.9 |
| Agree |  | 60.3 | 73.1 | 72.6 |
| Strongly agree |  | 20.7 | 21 | 21.5 |
| Respondent's school is a place where people trust them (%) | 32 |  |  |  |
| (Strongly) Disagree |  | 19.6 | 4.6 | 4.5 |
| Agree |  | 60.2 | 73.1 | 72.9 |
| Strongly agree |  | 20.3 | 22.3 | 22.6 |
| Respondent's school is a place where they know people who think a lot of me (%) | 46 |  |  |  |
| Strongly disagree |  | 18.1 | 3 | 3.2 |
| Disagree |  | 17 | 18.9 | 19.7 |
| Agree |  | 54 | 66.1 | 64.8 |
| Strongly agree |  | 11 | 12 | 12.3 |
| Respondent's school is a place where they have a lot of fun (%) | 31 |  |  |  |
| Strongly disagree |  | 17.5 | 2.7 | 2.7 |
| Disagree |  | 13.6 | 13.6 | 13.4 |
| Agree |  | 48.8 | 60.1 | 59.8 |
| Strongly agree |  | 20.2 | 23.6 | 24.1 |
| Respondent's school is a place where they really like to go each day (%) | 32 |  |  |  |
| Strongly disagree |  | 15.3 | 5.9 | 6.2 |
| Disagree |  | 25.7 | 29.9 | 28.9 |
| Agree |  | 47.1 | 57.7 | 57.5 |
| Strongly agree |  | 11.9 | 6.6 | 7.4 |
| Respondent's school is a place where they get excited about the work they d (%) | 35 |  |  |  |
| Strongly disagree |  | 14.3 | 12.8 | 12.8 |
| Disagree |  | 47.8 | 58.3 | 57 |
| Agree |  | 22.9 | 26.6 | 27.7 |
| Strongly agree |  | 14.9 | 2.3 | 2.5 |
| Respondent's school is a place where they enjoy what they do in class (%) | 34 |  |  |  |
| Strongly disagree |  | 14.2 | 2.3 | 2.7 |
| Disagree |  | 19.5 | 21.4 | 20.8 |
| Agree |  | 55.2 | 68.7 | 68.5 |
| Strongly agree |  | 11.1 | 7.6 | 8.1 |
| Sex (female = 1) | 0 | 0.54 (0.50) | 0.54 (0.50) | 0.49 (0.50) |
| Maternal smoking in pregnancy = 1 (%) | 6 | 18.7 | 18.3 | 25.2 |
| Home owner (including mortgage) = 1 (%) | 7 | 82.3 | 82.9 | 73.2 |
| Highest parental education (vocational) = 1 (%) | 17 | 7.2 | 5.2 | 5.8 |
| Highest parental education (O-level) = 1 (%) | 17 | 27.6 | 26.8 | 27.8 |
| Highest parental education (A-level) = 1 (%) | 17 | 36.4 | 38.1 | 35.5 |
| Highest parental education (Degree) = 1 (%) | 17 | 25.7 | 25.7 | 24.7 |
| Maternal experienced material hardship (mean (SD)) | 10 | 2.57 (3.34) | 2.51 (3.29) | 2.91 (3.54) |
| Parity (mean (SD)) | 8 | 0.78 (0.93) | 0.77 (0.92) | 0.84 (1.00) |
| Maternal age at birth (mean (SD)) | 11 | 29.31 (4.55) | 29.35 (4.53) | 28.64 (4.86) |
| WISC - Total IQ: F8 (mean (SD)) | 22 | 103.88 (16.42) | 105.01 (16.06) | 103.97 (16.53) |
| Capped GCSE point score (mean (SD)) | 0 | 344.67 (78.33) | 344.67 (78.33) | 318.94 (93.95) |
| Child enjoys school age 5 = Yes (%) | 0.29 | 84.7 | 87.5 | 86.7 |
| Child enjoys school age 6 = Yes (%) | 0.35 | 83.1 | 86.8 | 83.1 |

Table S3. Associations between child sex and exposures / outcomes

|  | **Child sex** |
| --- | --- |
| **T1: Ages 10-11** |  |
| Enjoyment | 0.17 (0.15, 0.19) |
| Connectedness | 0.10 (0.08, 0.12) |
| Externalising | -0.10 (-0.12, -0.08) |
| Depression | -0.06 (-0.08, -0.04) |
|  |  |
| **T2: Ages 13-14** |  |
| Enjoyment | -0.01 (-0.03, 0.01) |
| Connectedness | 0.13 (0.11, 0.15) |
| Externalising | -0.04 (-0.06, -0.02) |
| Depression | 0.03 (0.01, 0.05) |
|  |  |
| **T3: Age 16** |  |
| Attainment | 0.10 (0.08, 0.12) |

Table S4. School experience questionnaire items and factor loadings

|  | **Loading** |
| --- | --- |
| **T1: Ages 10 -11** |  |
| **Connectedness** |  |
| School is a place where other pupils accept me for who I am | 0.69 |
| School is a place where other pupils are very friendly | 0.81 |
| School is a place where people can depend upon me | 0.78 |
| School is a place where people trust me | 0.79 |
| School is a place where people think a lot of me | 0.73 |
| School is a place where I have a lot of fun | 0.82 |
| School is a place where I get on well with the other pupils | 0.82 |
|  |  |
| **Enjoyment** |  |
| School is a place where I really like to go each day | 0.75 |
| School is a place where I get excited about the work I do | 0.71 |
| School is a place where I enjoy what I do in class | 0.93 |
|  |  |
|  |  |
| **T2: Ages 13 - 14** |  |
| **Connectedness** |  |
| School is a place I get on well with other pupils | 0.92 |
| School is a place where other pupils accept me | 0.89 |
| School is a place where other pupils are very friendly | 0.91 |
| School is a place where people can depend on me | 0.75 |
| School is a place where people trust me | 0.83 |
| School is a place where I know people who think a lot of me | 0.82 |
| School is a place where I have a lot of fun | 0.85 |
|  |  |
| **Enjoyment** |  |
| School is a place where I really like to go each day | 0.66 |
| School is a place where I get excited about the work I do | 0.01 |
| School is a place where I enjoy what I do in class | 0.88 |

Table S5. Externalising factor loadings

|  | **Loading** |
| --- | --- |
|  |  |
| **T1: Ages 10-11** |  |
| Child has often had temper tantrums or hot tempers | 0.59 |
| Child has been generally obedient, usually does what adults request | 0.61 |
| Child has often fought with other children or bullied them | 0.68 |
| Child has often lied or cheated | 0.70 |
| Child has stolen from home/school/elsewhere | 0.70 |
| Child has been restless/overactive/cannot sit still for long | 0.76 |
| Child has been constantly fidgeting or squirming | 0.73 |
| Child has been easily distracted, concentration wandered | 0.80 |
| Child has thought things out before acting | 0.68 |
| Child has seen tasks through to the end, had good attention span | 0.77 |
|  |  |
| **T2: Ages 13-14** |  |
| Teenager is generally obedient, usually does what adults request | 0.65 |
| Teenager has often had temper tantrums or hot tempers | 0.65 |
| Teenager often fights or bullies other children/teenagers | 0.75 |
| Teenager often lies or cheats | 0.76 |
| Teenager steels from home, school, elsewhere | 0.75 |
| Teenager thinks things out before acting | 0.71 |
| Teenager sees tasks through to end, has good attention span | 0.78 |
| Teenager has been restless, overactive and can't stay still for long | 0.77 |
| Teenager is constantly fidgeting or squirming | 0.80 |
| Teenager is easily distracted, concentration wanders | 0.80 |

Questions phrased in reverse direction recoded prior to analysis

Table S6. Depressive symptoms factor loadings

| **Variable** | **Loading** |
| --- | --- |
|  |  |
| **T1: Ages 10-11** |  |
| I felt miserable | 0.73 |
| I didn’t enjoy anything | 0.69 |
| I felt tired, sat around | 0.62 |
| I felt very restless | 0.43 |
| I felt they I was no good | 0.93 |
| I cried a lot | 0.80 |
| I found it hard to think | 0.68 |
| I hated myself | 0.93 |
| I felt I was a bad person | 0.73 |
| I felt lonely | 0.85 |
| I thought nobody loved me | 0.93 |
| I felt I was not as good as others | 0.86 |
| I felt I did everything wrong | 0.82 |
|  |  |
| **T2: Ages 13-14** |  |
| I felt miserable | 0.83 |
| I didn’t enjoy anything | 0.79 |
| I felt tired, sat around | 0.72 |
| I felt very restless | 0.67 |
| I felt they I was no good | 0.97 |
| I cried a lot | 0.86 |
| I found it hard to think | 0.79 |
| I hated myself | 0.97 |
| I felt I was a bad person | 0.92 |
| I felt lonely | 0.93 |
| I thought nobody loved me | 0.97 |
| I felt I was not as good as others | 0.93 |
| I felt I did everything wrong | 0.95 |

Table S7. Within time-point residual correlations between school enjoyment, connectedness and externalising (n=6409)

|  | | ***Residual correlation*** |
| --- | --- | --- |
| **T1: Ages 10-11** | |  |
| Connectedness | Enjoyment | 0.73 (0.72, 0.74) |
| Externalising | Connectedness | -0.19 (-0.21, -0.17) |
| Externalising | Enjoyment | -0.11 (-0.13, -0.09) |
|  |  |  |
| **T2: Ages 13-14** | | |
| Connectedness | Enjoyment | 0.58 (0.56, 0.60) |
| Externalising | Connectedness | -0.15 (-0.17, -0.13) |
| Externalising | Enjoyment | -0.12 (-0.14, -0.10) |

Values are Pearson correlation coefficients with 95% confidence intervals. Correlations adjusted for adjusted for child sex, maternal smoking in pregnancy, housing tenure, (highest) parental education, self-report of material hardship, parity and school enjoyment at ages 5 and 6.

Table S8. Within time-point residual correlations between school enjoyment, connectedness and depressive symptoms (n=6409)

|  | | ***Residual correlation*** |
| --- | --- | --- |
|  | | |
| **T1: Ages 10-11** | | |
| Connectedness | Enjoyment | 0.73 (0.72, 0.74) |
| Depression | Connectedness | -0.26 (-0.28, -0.24) |
| Depression | Enjoyment | -0.13 (-0.15, -0.11) |
|  |  |  |
| **T2: Ages 13-14** | | |
| Connectedness | Enjoyment | 0.59 (0.57, 0.61) |
| Depression | Connectedness | -0.25 (-0.27, -0.23) |
| Depression | Enjoyment | -0.21 (-0.23, -0.19) |

Values are Pearson correlation coefficients with 95% confidence intervals. Correlations adjusted for adjusted for child sex, maternal smoking in pregnancy, housing tenure, (highest) parental education, self-report of material hardship, parity and school enjoyment at ages 5 and 6.

Table S9. Externalising Structural Equation Model Full Sample (n=6,409)

|  | **Unadjusted** | **Adjusted 1** | **Adjusted 2** |
| --- | --- | --- | --- |
| **T2 connectedness ~** |  |  |  |
| T1 connectedness | 0.14 (0.07, 0.21) | 0.15 (0.09, 0.22) | 0.15 (0.09, 0.22) |
| T1 enjoyment | 0.00 (-0.07, 0.07) | -0.04 (-0.10, 0.03) | -0.04 (-0.10, 0.03) |
| T1 externalising | -0.15 (-0.19, -0.11) | -0.13 (-0.17, -0.08) | -0.13 (-0.17, -0.08) |
| **T2 enjoyment ~** |  |  |  |
| T1 connectedness | -0.16 (-0.24, -0.07) | -0.15 (-0.23, -0.06) | -0.15 (-0.24, -0.07) |
| T1 enjoyment | 0.42 (0.34, 0.50) | 0.40 (0.32, 0.48) | 0.41 (0.33, 0.49) |
| T1 externalising | -0.10 (-0.16, -0.05) | -0.08 (-0.13, -0.03) | -0.07 (-0.12, -0.02) |
| **T2 externalising ~** |  |  |  |
| T1 connectedness | 0.04 (-0.02, 0.10) | 0.04 (-0.02, 0.10) | 0.04 (-0.02, 0.10) |
| T1 enjoyment | -0.05 (-0.11, 0.01) | -0.05 (-0.12, 0.01) | -0.05 (-0.11, 0.01) |
| T1 externalising | 0.73 (0.69, 0.76) | 0.72 (0.68, 0.75) | 0.72 (0.69, 0.76) |
| **T3 attainment ~** |  |  |  |
| T2 connectedness | 0.01 (-0.05, 0.06) | -0.01 (-0.06, 0.05) | 0.00 (-0.05, 0.05) |
| T2 enjoyment | 0.12 (0.06, 0.18) | 0.08 (0.03, 0.13) | 0.08 (0.03, 0.14) |
| T2 externalising | -0.20 (-0.25, -0.15) | -0.16 (-0.21, -0.12) | -0.11 (-0.16, -0.06) |
| T1 externalising | -0.20 (-0.25, -0.15) | -0.16 (-0.20, -0.12) | -0.12 (-0.16, -0.07) |

Values are standardised regression coefficients with 95% confidence intervals. Model “Adjusted 1” adjusted for child sex, maternal smoking in pregnancy, housing tenure, (highest) parental education, self-report of material hardship and parity and school enjoyment at ages 5 and 6. Model “Adjusted 2” additionally adjusted for child IQ. “T2” = ages 13-14; “T3” = age 16

Table S10. Depressive Symptoms Structural Equation Model Full Sample (n=6,409)

|  | **Unadjusted** | **Adjusted 1** | **Adjusted 2** |
| --- | --- | --- | --- |
| **T2 connectedness ~** |  |  |  |
| T1 connectedness | 0.14 (0.07, 0.21) | 0.16 (0.09, 0.23) | 0.16 (0.09, 0.23) |
| T1 enjoyment | 0.01 (-0.06, 0.07) | -0.04 (-0.10, 0.03) | -0.03 (-0.10, 0.04) |
| T1 depression | -0.08 (-0.13, -0.04) | -0.06 (-0.11, -0.01) | -0.05 (-0.10, -0.01) |
| **T2 enjoyment ~** |  |  |  |
| T1 connectedness | -0.16 (-0.25, -0.07) | -0.14 (-0.23, -0.05) | -0.15 (-0.23, -0.06) |
| T1 enjoyment | 0.43 (0.35, 0.51) | 0.40 (0.32, 0.48) | 0.41 (0.33, 0.49) |
| T1 depression | -0.06 (-0.12, -0.01) | -0.04 (-0.09, 0.02) | -0.03 (-0.08, 0.03) |
| **T2 depression ~** |  |  |  |
| T1 connectedness | 0.07 (0.01, 0.13) | 0.07 (0.00, 0.13) | 0.06 (0.00, 0.13) |
| T1 enjoyment | -0.06 (-0.12, 0.01) | -0.06 (-0.13, 0.01) | -0.06 (-0.13, 0.01) |
| T1 depression | 0.43 (0.37, 0.48) | 0.41 (0.36, 0.47) | 0.41 (0.35, 0.47) |
| **T3 attainment ~** |  |  |  |
| T2 connectedness | 0.03 (-0.04, 0.10) | 0.02 (-0.04, 0.08) | 0.01 (-0.04, 0.06) |
| T2 enjoyment | 0.15 (0.08, 0.22) | 0.10 (0.04, 0.15) | 0.10 (0.05, 0.15) |
| T2 depression | -0.06 (-0.10, -0.02) | -0.04 (-0.08, -0.01) | -0.05 (-0.08, -0.01) |
| T1 depression | -0.20 (-0.23, -0.16) | -0.14 (-0.17, -0.11) | -0.07 (-0.10, -0.04) |

Values are standardised regression coefficients with 95% confidence intervals. Model “Adjusted 1” adjusted for child sex, maternal smoking in pregnancy, housing tenure, (highest) parental education, self-report of material hardship and parity and school enjoyment at ages 5 and 6. Model “Adjusted 2” additionally adjusted for child IQ. “T2” = ages 13-14; “T3” = age 16

Table S11. Externalising Structural Equation Model: Males Only (n = 2,950)

|  | **Unadjusted** | **Adjusted 1** | **Adjusted 2** |
| --- | --- | --- | --- |
| **T2 connectedness ~** |  |  |  |
| T1 connectedness | 0.12 (0.02, 0.23) | 0.14 (0.03, 0.24) | 0.14 (0.03, 0.24) |
| T1 enjoyment | 0.00 (-0.11, 0.10) | -0.02 (-0.13, 0.08) | -0.02 (-0.12, 0.09) |
| T1 externalising | -0.16 (-0.21, -0.1) | -0.14 (-0.20, -0.08) | -0.14 (-0.20, -0.07) |
| **T2 enjoyment ~** |  |  |  |
| T1 connectedness | -0.30 (-0.67, 0.07) | -0.28 (-0.69, 0.13) | -0.28 (-0.65, 0.08) |
| T1 enjoyment | 0.54 (0.09, 1.00) | 0.51 (-0.01, 1.03) | 0.52 (0.08, 0.96) |
| T1 externalising | -0.21 (-0.46, 0.03) | -0.18 (-0.42, 0.05) | -0.15 (-0.34, 0.04) |
| **T2 externalising ~** |  |  |  |
| T1 connectedness | 0.01 (-0.06, 0.08) | 0.01 (-0.06, 0.08) | 0.01 (-0.06, 0.08) |
| T1 enjoyment | -0.01 (-0.09, 0.06) | -0.01 (-0.08, 0.07) | -0.01 (-0.09, 0.06) |
| T1 externalising | 0.81 (0.78, 0.84) | 0.80 (0.77, 0.82) | 0.79 (0.76, 0.82) |
| **T3 attainment ~** |  |  |  |
| T2 connectedness | 0.08 (-0.13, 0.30) | 0.06 (-0.12, 0.24) | 0.01 (-0.12, 0.15) |
| T2 enjoyment | 0.04 (-0.17, 0.24) | 0.03 (-0.14, 0.20) | 0.06 (-0.08, 0.21) |
| T2 externalising | -0.24 (-0.33, -0.16) | -0.16 (-0.24, -0.09) | -0.12 (-0.20, -0.05) |
| T1 externalising | -0.16 (-0.25, -0.08) | -0.17 (-0.25, -0.10) | -0.12 (-0.20, -0.05) |

Values are standardised regression coefficients with 95% confidence intervals. Model “Adjusted 1” adjusted for child sex, maternal smoking in pregnancy, housing tenure, (highest) parental education, self-report of material hardship and parity and school enjoyment at ages 5 and 6. Model “Adjusted 2” additionally adjusted for child IQ. “T2” = ages 13-14; “T3” = age 16

Table S12. Externalising Structural Equation Model: Females Only (n = 3,459)

|  | **Unadjusted** | **Adjusted 1** | **Adjusted 2** |
| --- | --- | --- | --- |
| **T2 connectedness ~** |  |  |  |
| T1 connectedness | 0.17 (0.07, 0.26) | 0.17 (0.07, 0.26) | 0.17 (0.07, 0.27) |
| T1 enjoyment | -0.03 (-0.13, 0.06) | -0.04 (-0.14, 0.05) | -0.05 (-0.14, 0.05) |
| T1 externalising | -0.14 (-0.19, -0.09) | -0.12 (-0.17, -0.06) | -0.12 (-0.17, -0.06) |
| **T2 enjoyment ~** |  |  |  |
| T1 connectedness | -0.10 (-0.21, 0.01) | -0.09 (-0.20, 0.02) | -0.10 (-0.21, 0.01) |
| T1 enjoyment | 0.33 (0.22, 0.43) | 0.31 (0.21, 0.42) | 0.32 (0.22, 0.43) |
| T1 externalising | -0.09 (-0.15, -0.03) | -0.05 (-0.11, 0.01) | -0.04 (-0.11, 0.02) |
| **T2 externalising ~** |  |  |  |
| T1 connectedness | 0.07 (-0.01, 0.15) | 0.07 (-0.01, 0.16) | 0.08 (-0.01, 0.16) |
| T1 enjoyment | -0.10 (-0.18, -0.01) | -0.09 (-0.18, 0.00) | -0.10 (-0.19, -0.01) |
| T1 externalising | 0.67 (0.62, 0.72) | 0.66 (0.61, 0.71) | 0.65 (0.59, 0.70) |
| **T3 attainment ~** |  |  |  |
| T2 connectedness | -0.08 (-0.14, -0.01) | -0.07 (-0.13, -0.01) | -0.05 (-0.10, 0.00) |
| T2 enjoyment | 0.21 (0.15, 0.27) | 0.15 (0.09, 0.21) | 0.13 (0.08, 0.18) |
| T2 externalising | -0.20 (-0.27, -0.14) | -0.17 (-0.22, -0.11) | -0.13 (-0.18, -0.07) |
| T1 externalising | -0.19 (-0.25, -0.12) | -0.15 (-0.20, -0.09) | -0.09 (-0.13, -0.04) |

Values are standardised regression coefficients with 95% confidence intervals. Model “Adjusted 1” adjusted for child sex, maternal smoking in pregnancy, housing tenure, (highest) parental education, self-report of material hardship and parity and school enjoyment at ages 5 and 6. Model “Adjusted 2” additionally adjusted for child IQ. “T2” = ages 13-14; “T3” = age 16

Table S13. Depressive Symptoms Structural Equation Model: Males Only (n = 2,950)

|  | **Unadjusted** | **Adjusted 1** | **Adjusted 2** |
| --- | --- | --- | --- |
| **T2 connectedness ~** |  |  |  |
| T1 connectedness | 0.14 (0.03, 0.25) | 0.15 (0.04, 0.26) | 0.15 (0.04, 0.26) |
| T1 enjoyment | 0.00 (-0.11, 0.10) | -0.02 (-0.13, 0.08) | -0.02 (-0.13, 0.09) |
| T1 depression | -0.09 (-0.16, -0.02) | -0.07 (-0.14, -0.01) | -0.07 (-0.14, 0.00) |
| **T2 enjoyment ~** |  |  |  |
| T1 connectedness | -0.30 (-0.74, 0.13) | -0.28 (-0.7, 0.15) | -0.27 (-0.64, 0.10) |
| T1 enjoyment | 0.55 (0.00, 1.10) | 0.50 (-0.04, 1.04) | 0.51 (0.06, 0.96) |
| T1 depression | -0.18 (-0.4, 0.04) | -0.14 (-0.34, 0.05) | -0.11 (-0.26, 0.04) |
| **T2 depression ~** |  |  |  |
| T1 connectedness | 0.12 (0.02, 0.22) | 0.10 (0.01, 0.20) | 0.10 (0.01, 0.20) |
| T1 enjoyment | -0.12 (-0.23, -0.02) | -0.11 (-0.22, -0.01) | -0.11 (-0.21, -0.01) |
| T1 depression | 0.46 (0.39, 0.53) | 0.45 (0.38, 0.52) | 0.45 (0.37, 0.52) |
| **T3 attainment ~** |  |  |  |
| T2 connectedness | 0.09 (-0.16, 0.35) | 0.09 (-0.11, 0.29) | 0.02 (-0.15, 0.19) |
| T2 enjoyment | 0.08 (-0.18, 0.33) | 0.04 (-0.16, 0.24) | 0.09 (-0.10, 0.27) |
| T2 depression | -0.05 (-0.11, 0.01) | -0.02 (-0.08, 0.03) | -0.01 (-0.07, 0.04) |
| T1 depression | -0.19 (-0.24, -0.15) | -0.16 (-0.2, -0.12) | -0.10 (-0.15, -0.06) |

Values are standardised regression coefficients with 95% confidence intervals. Model “Adjusted 1” adjusted for child sex, maternal smoking in pregnancy, housing tenure, (highest) parental education, self-report of material hardship and parity and school enjoyment at ages 5 and 6. Model “Adjusted 2” additionally adjusted for child IQ. “T2” = ages 13-14; “T3” = age 16

Table S14. Depressive Symptoms Structural Equation Model: Females Only (n = 3,459)

|  | **Unadjusted** | **Adjusted 1** | **Adjusted 2** |
| --- | --- | --- | --- |
| **T2 connectedness ~** |  |  |  |
| T1 connectedness | 0.17 (0.07, 0.26) | 0.17 (0.08, 0.27) | 0.17 (0.08, 0.27) |
| T1 enjoyment | -0.03 (-0.13, 0.07) | -0.04 (-0.14, 0.05) | -0.04 (-0.14, 0.05) |
| T1 depression | -0.07 (-0.14, 0.00) | -0.05 (-0.12, 0.02) | -0.05 (-0.12, 0.02) |
| **T2 enjoyment ~** |  |  |  |
| T1 connectedness | -0.11 (-0.23, 0.00) | -0.09 (-0.21, 0.02) | -0.10 (-0.22, 0.01) |
| T1 enjoyment | 0.34 (0.24, 0.45) | 0.32 (0.22, 0.43) | 0.33 (0.23, 0.44) |
| T1 depression | -0.06 (-0.14, 0.01) | -0.02 (-0.1, 0.05) | -0.01 (-0.09, 0.07) |
| **T2 depression ~** |  |  |  |
| T1 connectedness | 0.02 (-0.06, 0.11) | 0.02 (-0.06, 0.11) | 0.02 (-0.06, 0.11) |
| T1 enjoyment | -0.02 (-0.11, 0.06) | -0.01 (-0.1, 0.08) | -0.01 (-0.10, 0.08) |
| T1 depression | 0.41 (0.34, 0.47) | 0.38 (0.30, 0.45) | 0.38 (0.30, 0.45) |
| **T3 attainment ~** |  |  |  |
| T2 connectedness | -0.05 (-0.12, 0.02) | -0.04 (-0.10, 0.02) | -0.03 (-0.09, 0.02) |
| T2 enjoyment | 0.22 (0.15, 0.28) | 0.15 (0.09, 0.21) | 0.14 (0.08, 0.19) |
| T2 depression | -0.09 (-0.15, -0.03) | -0.07 (-0.12, -0.02) | -0.08 (-0.12, -0.03) |
| T1 depression | -0.18 (-0.23, -0.13) | -0.12 (-0.17, -0.08) | -0.04 (-0.08, 0.00) |

Values are standardised regression coefficients with 95% confidence intervals. Model “Adjusted 1” adjusted for child sex, maternal smoking in pregnancy, housing tenure, (highest) parental education, self-report of material hardship and parity and school enjoyment at ages 5 and 6. Model “Adjusted 2” additionally adjusted for child IQ. “T2” = ages 13-14; “T3” = age 16

Table S15. Externalising Complete Case Structural Equation Model (Unadjusted; n = 1,787)

| **T2 connectedness ~** |  |
| --- | --- |
| T1 connectedness | 0.48 (0.38, 0.58) |
| T1 enjoyment | -0.16 (-0.26, -0.05) |
| T1 externalising | -0.09 (-0.14, -0.03) |
| **T2 enjoyment ~** |  |
| T1 connectedness | 0.01 (-0.10, 0.12) |
| T1 enjoyment | 0.39 (0.29, 0.50) |
| T1 externalising | -0.13 (-0.19, -0.07) |
| **T2 externalising ~** |  |
| T1 connectedness | -0.02 (-0.10, 0.06) |
| T1 enjoyment | 0.02 (-0.06, 0.10) |
| T1 externalising | 0.82 (0.79, 0.84) |
| **T3 attainment ~** |  |
| T2 connectedness | -0.16 (-0.23, -0.09) |
| T2 enjoyment | 0.13 (0.06, 0.20) |
| T2 externalising | -0.12 (-0.21, -0.03) |
| T1 externalising | -0.31 (-0.39, -0.22) |

Values are standardised regression coefficients with 95% confidence intervals. Note only unadjusted results presented as model did not converge for complete case analysis when including covariates. “T2” = ages 13-14; “T3” = age 16

Table S16. Depressive Symptoms Complete Case Structural Equation Model (Unadjusted; n = 1,742)

| **T2 connectedness ~** |  |
| --- | --- |
| T1 connectedness | 0.49 (0.37, 0.61) |
| T1 enjoyment | -0.12 (-0.24, -0.01) |
| T1 depression | 0.00 (-0.06, 0.07) |
| **T2 enjoyment ~** |  |
| T1 connectedness | -0.07 (-0.19, 0.06) |
| T1 enjoyment | 0.46 (0.35, 0.58) |
| T1 depression | -0.07 (-0.14, 0.00) |
| **T2 depression ~** |  |
| T1 connectedness | -0.06 (-0.21, 0.08) |
| T1 enjoyment | 0.05 (-0.08, 0.18) |
| T1 depression | 0.31 (0.24, 0.38) |
| **T3 attainment ~** |  |
| T2 connectedness | -0.17 (-0.24, -0.10) |
| T2 enjoyment | 0.23 (0.16, 0.30) |
| T2 depression | 0.08 (0.02, 0.13) |
| T1 depression | -0.20 (-0.25, -0.14) |

Values are standardised regression coefficients with 95% confidence intervals. Note only unadjusted results presented as model did not converge for complete case analysis when including covariates. “T2” = ages 13-14; “T3” = age 16

Table S17. Hyperactivity Subscale Structural Equation Model Full Sample (n = 6,409)

|  | **Unadjusted** | **Adjusted 1** | **Adjusted 2** |
| --- | --- | --- | --- |
| **T2 connectedness ~** |  |  |  |
| T1 connectedness | 0.15 (0.08, 0.21) | 0.16 (0.09, 0.23) | 0.16 (0.09, 0.23) |
| T1 enjoyment | 0.00 (-0.07, 0.07) | -0.04 (-0.11, 0.03) | -0.04 (-0.11, 0.03) |
| T1 hyperactivity | -0.13 (-0.17, -0.09) | -0.10 (-0.15, -0.06) | -0.10 (-0.14, -0.06) |
| **T2 enjoyment ~** |  |  |  |
| T1 connectedness | -0.15 (-0.24, -0.07) | -0.14 (-0.23, -0.06) | -0.15 (-0.23, -0.06) |
| T1 enjoyment | 0.41 (0.34, 0.49) | 0.40 (0.32, 0.48) | 0.41 (0.32, 0.49) |
| T1 hyperactivity | -0.09 (-0.14, -0.04) | -0.07 (-0.11, -0.02) | -0.06 (-0.10, -0.01) |
| **T2 hyperactivity ~** |  |  |  |
| T1 connectedness | 0.04 (-0.02, 0.09) | 0.04 (-0.02, 0.10) | 0.04 (-0.02, 0.10) |
| T1 enjoyment | -0.05 (-0.11, 0.01) | -0.05 (-0.11, 0.01) | -0.06 (-0.12, 0.01) |
| T1 hyperactivity | 0.75 (0.72, 0.78) | 0.73 (0.7, 0.76) | 0.72 (0.69, 0.76) |
| **T3 attainment ~** |  |  |  |
| T2 connectedness | 0.01 (-0.04, 0.07) | 0.00 (-0.05, 0.05) | 0.00 (-0.05, 0.05) |
| T2 enjoyment | 0.11 (0.06, 0.17) | 0.08 (0.03, 0.13) | 0.08 (0.04, 0.13) |
| T2 hyperactivity | -0.23 (-0.29, -0.18) | -0.18 (-0.23, -0.13) | -0.14 (-0.18, -0.09) |
| T1 hyperactivity | -0.19 (-0.24, -0.13) | -0.16 (-0.21, -0.11) | -0.10 (-0.14, -0.06) |

Values are standardised regression coefficients with 95% confidence intervals. Model “Adjusted 1” adjusted for child sex, maternal smoking in pregnancy, housing tenure, (highest) parental education, self-report of material hardship and parity and school enjoyment at ages 5 and 6. Model “Adjusted 2” additionally adjusted for child IQ. “T2” = ages 13-14; “T3” = age 16

Table S18. Conduct Subscale Structural Equation Model Full Sample (n = 6,409)

|  | **Unadjusted** | **Adjusted 1** | **Adjusted 2** |
| --- | --- | --- | --- |
| **T2 connectedness ~** |  |  |  |
| T1 connectedness | 0.13 (0.07, 0.20) | 0.15 (0.08, 0.22) | 0.15 (0.08, 0.22) |
| T1 enjoyment | 0.01 (-0.06, 0.08) | -0.03 (-0.10, 0.04) | -0.03 (-0.10, 0.04) |
| T1 conduct | -0.16 (-0.21, -0.12) | -0.15 (-0.19, -0.10) | -0.15 (-0.19, -0.10) |
| **T2 conduct ~** |  |  |  |
| T1 connectedness | -0.16 (-0.24, -0.07) | -0.14 (-0.23, -0.06) | -0.15 (-0.24, -0.07) |
| T1 enjoyment | 0.43 (0.35, 0.50) | 0.40 (0.32, 0.48) | 0.41 (0.33, 0.50) |
| T1 conduct | -0.11 (-0.17, -0.05) | -0.08 (-0.14, -0.02) | -0.07 (-0.14, -0.01) |
| **T2 conduct ~** |  |  |  |
| T1 connectedness | 0.05 (-0.01, 0.12) | 0.06 (-0.01, 0.12) | 0.06 (-0.01, 0.13) |
| T1 enjoyment | -0.05 (-0.12, 0.02) | -0.06 (-0.13, 0.01) | -0.06 (-0.13, 0.01) |
| T1 conduct | 0.74 (0.70, 0.78) | 0.73 (0.68, 0.77) | 0.72 (0.68, 0.77) |
| **T3 attainment ~** |  |  |  |
| T2 connectedness | 0.01 (-0.06, 0.08) | 0.00 (-0.06, 0.06) | -0.01 (-0.06, 0.05) |
| T2 enjoyment | 0.15 (0.08, 0.22) | 0.10 (0.04, 0.16) | 0.10 (0.05, 0.15) |
| T2 conduct | -0.13 (-0.20, -0.06) | -0.11 (-0.17, -0.05) | -0.10 (-0.15, -0.04) |
| T1 conduct | -0.16 (-0.23, -0.09) | -0.12 (-0.17, -0.06) | -0.07 (-0.13, -0.02) |

Values are standardised regression coefficients with 95% confidence intervals. Model “Adjusted 1” adjusted for child sex, maternal smoking in pregnancy, housing tenure, (highest) parental education, self-report of material hardship and parity and school enjoyment at ages 5 and 6. Model “Adjusted 2” additionally adjusted for child IQ. “T2” = ages 13-14; “T3” = age 16
